# Time to tweak the TTO: results from a comparison of alternative specifications of the TTO

**DOI:** 10.1007/s10198-013-0507-y

**Published:** 2013-07-31

**Authors:** Matthijs M. Versteegh, Arthur E. Attema, Mark Oppe, Nancy J. Devlin, Elly A. Stolk

**Affiliations:** 1 iMTA/iBMG, Institute of Health Policy and Management/Institute for Medical Technology Assessment, Erasmus University of Rotterdam, PO Box 1738, 3000 DR Rotterdam, The Netherlands; 2 Office of Health Economics, London, UK

**Keywords:** Time trade-off, Lead-time TTO, Lag-time TTO, Utility, Health-state preferences, I10

## Abstract

This article examines the effect that different specifications of the time trade-off (TTO) valuation task may have on values for EQ-5D-5L health states. The new variants of the TTO, namely lead-time TTO and lag-time TTO, along with the classic approach to TTO were compared using two durations for the health states (15 and 20 years). The study tested whether these methods yield comparable health-state values. TTO tasks were administered online. It was found that lag-time TTO produced lower values than lead-time TTO and that the difference was larger in the longer time frame. Classic TTO values most resembled those of the lag-time TTO in a 20-year time frame in terms of mean absolute difference. The relative importance of different domains of health was systematically affected by the duration of the health state. In the tasks with a 10-year health-state duration, anxiety/depression had the largest negative impact on health-state values; in the tasks with a 5-year duration, the pain/discomfort domain had the largest negative impact.

## Introduction

Attempts to improve the measurement of health-state values have led to several methodological innovations in valuation techniques such as the time trade-off (TTO), which are used to determine the desirability of a hypothetical state of health. Novel specifications of the classic approach to TTO have been developed to make the measurement of health states considered ‘worse than dead’ (WTD) more accurate [1]. Lead-time TTO and lag-time TTO are in theory equally capable of addressing issues in the valuation of WTD health states. However, there is little evidence on how these methods compare. To help fill that gap, the classic approach to TTO (here referred to as ‘classic TTO’) and two novel methods (lead-time TTO and lag-time TTO) have been compared in an online study.

In the TTO, a value can be assigned to a health state by letting respondents trade off length of life against quality of life. The resulting value is generally taken to reflect the health-related quality of life per period for the duration of the health state. Classic TTO applies two different procedures for the valuation of health states that are considered better than dead and those considered WTD. Therefore, TTO values for health states better than dead and WTD may not lie on the same utility scale [2]. Furthermore, sacrificing one additional year in the WTD procedure lowers the value of a health state more than when 1 year is sacrificed in the better than dead procedure [3]. While values for health states better than dead are restricted to points between 0 and 1, the values measured with the procedure for WTD can become very low [4, 5]. Therefore, an arbitrary transformation of those values is subsequently needed to avoid distortion of the mean value.

The two alternative specifications of TTO do not have the above-mentioned limitations of the classic version. In lead-time TTO, first proposed by Robinson and Spencer [6] and extensively discussed elsewhere, the impaired health state ‘begins’ after a period of healthy years (the lead-time) [4]. In lag-time TTO, healthy life years *follow* the impaired health state rather than *preceding* it [7, 8]. Probably the most important application of either lead-time TTO or lag-time TTO is in the valuation of health state descriptive systems, such as EQ-5D.

In lead-time TTO, the health state under valuation is further away in the future than in lag-time TTO, where the health state ‘begins’ immediately. It could be hypothesized, therefore, that lead-time values for the same health state will be higher than lag-time values if respondents have positive time preferences, as frequently observed [9, 10], although there are also reports of negative time preferences for TTO [11]. Alternatively, it could be hypothesized that lag-time TTO results in higher values, since the lag-time of full health after a given health state might be interpreted as having been cured, which, arguably, influences the perception of the severity of the health state. Conceptually, lag-time TTO might be more ‘plausible’ for mild states and curative treatments, since it is based on the premise that poor health is followed by good health. Lead-time TTO may be more plausible for very severe health states and preventive treatments since it poses that the health state starts in the future and is followed by death [8].

In this study, respondents participated in an online experiment where they engaged in either lead-time TTO, lag-time TTO, or classic TTO. The purpose was to see how the health-state values produced by each of the TTOs compare. It also investigated how both the type of TTO and the duration of a health state would affect the values for each of the EQ-5D domains of health. Values generated by the online mode of administration were compared to values estimated on the basis of a face-to-face TTO. Scores on the respondents’ engagement with and understanding of the task were used to explain potential differences.

## Methods

### Respondents

A sample of respondents was drawn from members of a commercial panel. Only persons between 18 and 65 years of age were approached to participate in the online experiment. Stratification to represent the Dutch population was based on gender, education, and age. Respondents were not given a financial reward for participating.

### Health-state selection and description

Health states were based on the Dutch version of the five-level EQ-5D (EQ-5D-5L) [12]. This instrument consists of five domains of health: mobility, self-care, usual activities, pain/discomfort, and anxiety/depression. The instrument has five answer categories for each domain, generating 3,125 (5^5^) health states. Out of the total of 3,125 possible health states, 100 were selected in light of a previously developed D-optimal design [13].

### Study design

All respondents performed a combination of tasks. First, they filled out a background questionnaire. They also indicated how they perceived their own health on the EQ-5D-5L instrument and the EQ-5D visual analog scale. Scores on the latter ranged from 0 to 100, where 0 stood for the worst imaginable health and 100 the best imaginable. Then the respondents had to choose which of two EQ-5D-5L health states they considered best in a paired comparison task. Upon completing these preliminary tasks, the respondents were randomized over five different specifications of TTO: lead-time TTO with a duration of 15 years and of 20 years; lag-time TTO with a duration of 15 years and of 20 years; and classic TTO with a duration of 10 years. Within these five specifications, respondents were randomized over ten blocks containing ten EQ-5D-5L health states, and each state was presented in random order. The study ended with a short feasibility questionnaire.

#### The TTO tasks

In classic TTO, health-state values are elicited by asking respondents if they would prefer living *x* years in a period of full health to living *t* years in impaired health where *x* < *t*. If respondents accept living a shorter period *x* in full health, they are essentially willing to trade length of life for quality of life. The health-state value is then given by *x/t*, at the point of indifference. When the respondents would rather trade off all healthy life years than have to live in a particular health state for period *t*, they indicate that this health state is worse than dead (WTD), at least when the duration of that health state is equal to period *t*. Respondents then enter a different task to measure their negative preference values (since *x* < 0). In this WTD task, they are asked to choose between immediate dead and a life of duration *t*, with *x* years in full health preceded by *t*−*x* years in the imperfect health state. The value for the health state following this WTD task is generally −*x*/(*t*−*x*). In lead (or lag) time TTO, they were also asked if they would prefer living *x* years in full health compared to living *t* years in impaired health preceded (or followed) by *l* years in full health. An indifferent point was estimated by repeating this question for different values of *x*. The value of the health state is then given by (*x*−*l*)/*t*, where *x* is the estimated indifference value. When *x* < *l*, the formula results in a negative value, implying that these are WTD health states.

The TTO tasks were preceded by an animated instructional video. It explained how to trade off life years by giving an example with a hypothetical EQ-5D state, whereby an animated figure of a ‘doctor’ pointed out the various elements of the task. The video was designed to highlight the characteristics of the different TTO tasks. Thus, the examples shown in each animation preceding the real TTO task were identical in characteristics and layout to the real TTO task that followed, with the exception that the health state that was presented was not used in the study.

The classic TTO is a two-part task. The visual design and the health-state value equations for health states better than dead are different from those for WTD health states. The other four TTO tasks have a uniform visual representation and health state value equations for better than dead and WTD valuations. In all tasks, respondents are asked to choose between a fixed period in Life A and a variable period *t* in Life B. The value of *x* depends on the respondents’ previous choice for either Life A or B and follows the fixed iteration procedure described below.

#### Iteration procedure

The first two ‘steps’ of the fixed iteration procedure were similar for all five TTO tasks. At the first iteration, respondents were asked to choose between two scenarios: Life A, which contained the health state and, depending on the task, a lead-time or lag-time in full health, and Life B, which was set at the maximum of all years in full health (health-state value = 1, or *x* = 10, 15, or 20, depending on the total time frame). At the second iteration, the health-state value of Life B was 0 (or *x* = 0 for the classic TTO and *x* = 10 for the other variants). If respondents preferred Life A at value = 0, they would indicate that the health state is WTD. If they preferred Life B, they would indicate that the health state is better than dead. After this ‘sorting question,’ the iteration procedure continued with a choice between Life B and Life A where the value of B was set at *x* for value = 0.5 or −0.5. Conditional on choosing Life A or B, the remaining iterations represented value increments or decrements of 0.1 or 0.05 with the corresponding values of *x* in Life B.

#### Health-state value equations

The equations applied for the lead-time TTO in a 20-year time frame are (without discounting):

1 $$ 10U_{FH} + 10U_{{HS_{i} }} = xU_{FH} $$

where *U*_*FH*_ is the value (utility) of full health, *U*_*HSi*_ the value of the health state *i,* and *x* the number of years in full health at which the respondent indicated being indifferent in the TTO task. Solving for *U*_*HSi*_ gives:

2 $$ U_{{HS_{i} }} = \frac{x - 10}{10} $$

For a respondent who considers *x* = 13 years in full health equal to 10 years in full health followed by 10 years in health state *i*, the value for *i* is: *U*_*HSi*_ = (13−10)/10 = 0.3. In the same vein, the equation for lag-time TTO is:

3 $$ 10U_{{HS_{i} }} + 10U_{FH} = xU_{FH} $$

Equation 3 can also be solved for *U*_*HSi*_, which again results in Eq. 2. The most relevant details of the TTO specifications included in this study are described in the “Appendix” to enable easy comparison with other studies performed with a TTO checklist [14].

### Analysis

All respondents who completed the online exercise were included in the analyses. To check for consistency in findings, the analyses were rerun in a smaller sample without those respondents who: (1) indicated on the feasibility questionnaire that they did not understand the task; (2) did not differentiate among any of the ten health states; or (3) had used only three or fewer iterations for all health states.

#### Comparison of health-state values

Mean lead-time TTO and lag-time TTO values were compared for all 100 health states. The different minimum health-state values set for the TTO methods distort comparisons of the mean values between tasks. For example, solving the equations for *t* = 0 (trading in all life years) results in *U* = −2 for a ratio of lead-time to disease time of 2:1 and *U* = −1 for a ratio of 1:1. Therefore, comparisons of the mean are only made for tasks with similar attainable health-state values. Convergence of lead-time TTO and lag-time TTO with classic TTO was measured in terms of the mean absolute difference (MAD) to get a feel for the comparability of values despite the different ranges of health-state values.

The relative importance of the domains of EQ-5D in the different specifications of TTO is compared through random effects regression analysis to take account of the panel structure of the data (multiple TTO observations per respondent). Although the sizes of the coefficients are not directly comparable because of different ranges of the dependent variable (the TTO values), the relative importance of the domains within each regression model can still be compared. Independent variables in the regression model were the EQ-5D health domains, applied as continuous variables.

The online mode of administration of the TTO is still in an experimental stage. Also, the health-state values generated by the different tasks cannot be compared to a non-experimental EQ-5D-5L tariff, as the valuation protocol of the EQ-5D-5L was still under development at the time of this study. To get an indication of the convergent validity of the values produced in the online exercise, these values were compared to the estimated EQ-5D-5L values derived from a mapping function [15]. These estimates reveal which health-state value is expected for an EQ-5D-5L health state on the grounds of previous valuations for the EQ-5D-3L applied in face-to-face TTO.

#### Task engagement and response characteristics

Agreement among respondents in the different TTO tasks was ascertained with Levene’s test and Brown and Forsythe tests. It was assumed that differences in valuations between respondents, regardless of the cause, would result in greater variance and thus a less precise health-state value estimate. Although larger standard deviations may reflect preference heterogeneity rather than poorer task engagement, a valuation method that is identical in all respects but the onset of the health state (i.e., before or after a period of full health) is arguably preferable if there is more agreement among respondents. Variances for classic TTO (with transformed negative values) were only compared to the TTO tasks with a 20-year time frame, as TTO values for these two lie on the same −1 to 1 scale. Accordingly, the variances were not compared to values from the TTO tasks with a 15-year time frame (with a lead time to disease time ratio of 2:1), which lie on a −2 to 1 scale and thus logically have larger variances. Standard deviations, which lend themselves to a more intuitive interpretation than variances, were plotted for lead-time TTO and lag-time TTO. Other indicators of task engagement were used as well: whether the respondents were willing to trade off any time at all (non-traders); how many iterations the respondents used before reaching their point of indifference; how many respondents ‘used up’ all tradable time; and how many did not differentiate between health states.

#### Feasibility

Differences between tasks were compared using four items of a feasibility questionnaire presented after the TTO task. Respondents were asked to indicate their level of agreement with four statements: (1) The instructions that were given made it clear what I needed to do; (2) it was easy to understand the questions I was asked; (3) I found it difficult to decide on the exact point where Life A and B were about the same; (4) I found it easy to tell the difference between the health states I was asked to think about. The answer categories ranged from 1 (completely agree) to 5 (completely disagree). The mode, median, and percentiles of the answers on these questions were compared.

Since health-state values have been shown to be affected by the number of health states valued by a respondent, we repeated our analysis using only the first five valued health states [16]. We tested for significance of order effects by regressing the sequence of a health state on the number of iterations using ordinary least squares (OLS) regression, as proposed by Augestad et al. [16]. All statistical analyses were run in STATA 11.

## Results

In total, 5,208 respondents finished all the tasks, with approximately 1,000 respondents per task. The resulting data set was a balanced panel with 10 TTO observations for each respondent. Respondents in the online panel were slightly older than the Dutch population average [*mean* = 42.3 (SD = 14.2) versus Dutch population mean of 2009 = 40.1]. Furthermore, the panel contained more females, with 58.3 percent female and 41.7 percent male, compared to a nearly 50/50 distribution in the Netherlands. Mean self-assessed health on the EQ-5D visual analog scale (VAS) was 76.7 (SD = 17.4). Regression analysis indicated that respondents used fewer iterations (*p* < 0.001) for health states presented later in the sequence; on average, they used 0.4 iterations less than the previous health state for each consecutive one. Therefore, where relevant, results were rerun using only the first five health states.

### Comparison of health-state values

**Fig. 1:**
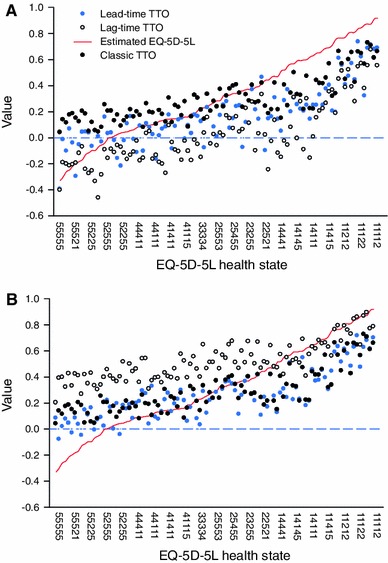
A comparison of classic TTO, lead-time TTO, lag-time TTO, and mapped EQ-5D-5L values

Lead-time TTO resulted in systematically higher values than lag-time TTO for the 20-year time frame (on average 0.25 higher) with larger average differences for poorer health states (Fig. 1a, b). In the 20-year time frame, none of the lag-time values were higher than the lead-time values. Results for the 15-year time frame were mixed: on average, lead-time TTO values were 0.13 higher in the 15-year time frame and lower than lag-time TTO values for 18 out of 100 health states (28 out of 100 using the first five health states). In terms of mean absolute deviation (MAD), differences between classic TTO and the other specifications (from least to most) were as follows: lag-time TTO in a 20-year time frame (MAD = 0.07); lead-time TTO in a 15-year time frame (MAD = 0.14); lead-time TTO in a 20-year time frame (MAD = 0.23); and lag-time TTO in a 15-year time frame (MAD = 0.26).

The range of health-state values in the 15-year time frame was 1.13 for lead-time TTO (from −0.4 to 0.73) and 1.14 for lag-time TTO (−0.46 to 0.68). In the 20-year time frame, values were higher than in the 15-year time frame for both variants. The higher health-state value was most likely due to the range of attainable values in the 20-year time frame (the minimum value of the 15-year time frame was −2, compared to −1 in the 20-year time frame. The minimum value of −1 also influenced the observed range of values in the 20-year time frame, which was smaller for both variants: the range was 0.69 for lead-time TTO (0.20–0.89) and 0.80 for lag-time TTO (−0.08 to 0.72). As can be seen in Fig. 1a, b, the range of values produced by the lead-time TTO and the lag-time TTO was smaller than would be expected in view of the estimated EQ-5D-5L values. Classic TTO, the method used for EQ-5D-3L, also produced a range that was smaller than expected (0.69). The worst health-state value^1^ with classic TTO was 0.04 (for state 55555), and the best was 0.73 (for 12111).

The specification of the TTO task influenced the relative importance of the different domains of health (Table 1). The size of the regression coefficients represents the marginal decrement in health-state values caused by scoring one point higher in a particular domain on the five-level descriptive system. The order of their relative importance was not affected by the choice for lead-time TTO or lag-time TTO but by the duration of the health state. In the 20-year time frames, with a disease duration of 10 years, the health domain ‘anxiety/depression’ was considered worse than ‘pain/discomfort.’ The inverse was found for the 15-year time frame, which has a disease duration of 5 years. Similarly, problems in usual activities were considered more problematic than problems with self-care in the 20-year time frame while the inverse was found for the 15-year time frame. The order in the classic TTO was different from the order in the lead-time TTO and lag-time TTO. The regression models using only the first five health states gave orderings that were identical to those found using all ten health states.

**Table 1:** Relative importance of different domains of health at different durations

	Classic TTO		15-year lead-time TTO		20-year lead-time TTO		15-year lag-time TTO		20-year lag-time TTO	
	Coef.	Imp.	Coef.	Imp.	Coef.	Imp.	Coef.	Imp.	Coef.	Imp.
Mobility	−0.026	3	−0.032	3	−0.026	3	−0.039	3	−0.036	3
Self-care	−0.020	1	−0.027	1	−0.020	2	−0.033	1	−0.028	2
Usual activities	−0.022	2	−0.028	2	−0.020	1	−0.038	2	−0.019	1
Pain/discomfort	−0.040	4	−0.057	5	−0.031	4	−0.060	5	−0.043	4
Anxiety/depression	−0.043	5	−0.053	4	−0.040	5	−0.058	4	−0.045	5
Constant	0.731		0.740		0.915		0.692		0.751	
Adjusted R-square	0.12		0.13		0.10		0.12		0.13	

All coefficients *p* < 0.05

*Imp.* relative importance

### Task engagement and response characteristics

**Fig. 2:**
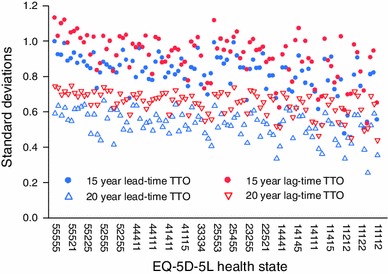
Standard deviations of two specifications of lead-time TTO and lag-time TTO

Lag-time TTO showed a larger variance than lead-time TTO for nearly all health states (Fig. 2). The mean variance of lag-time TTO is higher in both the 15-year time frame (*p* < 0.001) and the 20-year time frame (*p* < 0.001). The classic TTO with transformed negative values has a smaller variance than lag-time TTO (*p* < 0.001) but a larger variance than lead-time TTO (*p* < 0.001). When including only those respondents who had indicated on the feasibility questionnaire that they thought the task was clear (answer 1 to question 1), that they understood the task (answer 1 to question 2), or had not valued all ten health states equally, all statistical tests indicated significant differences (*p* < 0.001). A mean standard deviation of 0.81 (*N* = 1,067) was found for the online lead-time TTO in a 15-year time frame. When including only those respondents who were randomized to the LT-TTO in a 15-year time frame and who indicated they thought the task was clear and understood the task, the mean standard deviation increased somewhat to 0.83 (*N* = 359). Using only the first five valued health states increased the mean standard deviation of the lead-time TTO in a 15-year time frame to 0.84 (*N* = 533).

The number of non-traders (percentage health-state value = 1) and the distribution of better than dead (health-state value >0) and WTD (health-state value <0) responses suggest that the lead-time TTO causes respondents to judge health states as being less severe compared to lag-time TTO (Table 2). Interestingly, a large percentage of the respondents valued a state as equal to being dead (health-state value = 0). Also, more than 60 % of the respondents used only four iterations or less and about 35 % of the sample valued health states at value = 1. The median number of iterations was three for all specifications of TTO.

**Table 2:** Response characteristics

	Health state value = 1	Health state value = 0	Health state value < 0	Lowest value	No differentiation between 10 health states	Respondents using 4 or fewer iterations
Classic TTO	29.8	21.8	23.9	3.8	11.1	64.6
15-Year lead-time TTO	31.4	22.2	25.7	2.3	11.5	65.5
20-Year lead-time TTO	39.7	13.2	12.7	2.1	13.4	63.8
15 -Year lag-time TTO	33.5	17.3	35.7	3.5	10.6	65.5
20-Year lag-time TTO	32.8	18.5	29.2	4.2	10.8	64.8

### Feasibility

The mode response on statements 1, 2, and 3 of the feasibility questionnaire was ‘completely agree’ in all five specifications of TTO. For statement 4 (‘I found it easy to tell the difference between the health states I was asked to think about’), the mode response was ‘neutral,’ which again was similar for all specifications of TTO. Answer distributions differed for statements 1, 2, and 4 (Kruskall-Wallis test, *p* < 0.001) but were similar for statement 3 (‘I found it difficult to decide on the exact point where Life A and Life B were about the same’) (Kruskal-Wallis test, *p* = 0.43). For statements 1, 2, and 4, the lead-time TTO in a 20-year time frame was systematically considered slightly more difficult. No clear patterns were discerned between feasibility statements and gender or health of the respondents as measured by EuroQol-VAS.

## Discussion

In this study, classic TTO and novel specifications of the TTO method were compared to explore the impact of the specifications of the task on health-state values. The specifications of the TTO tasks applied in this study systematically affected health-state values and the relative importance of domains of health. In the 20-year time frame, lag-time TTO produced lower values than lead-time TTO, but the results for the 15-year time frame were mixed. Classic TTO values with transformed negative values most resembled those from lag-time TTO in a 20-year time frame. The relative importance of different domains of health was affected by the duration of the impaired health state, but not by the choice for lead-time TTO or lag-time TTO. It appears that respondents considered anxiety/depression to be worse than pain/discomfort only for a duration longer than 5 years.

Lag-time TTO resulted in lower values than lead-time TTO, and this effect was most pronounced in the 20-year time frame. On average, the effect of time preference (i.e., preferring to be in the best health state immediately) on health-state values is larger than the ‘preference for improvement’ effect (i.e., that the bad health state will be followed by a good health state). From these findings, it seems that the additive separability assumption of the QALY model (i.e., a health-state value is independent of the health states preceding or following it) does not hold, as health-state values elicited with lag-time TTO are lower than those found with lead-time TTO. We are only aware of one previously published study testing lag-time TTO [8]. In that study, lag-time TTO did not produce the same values as lead-time TTO using seven EQ-5D health states. In the present study, which used 5-year disease time and 10-year lead/lag-time, lead-time TTO values were lower for more severe states than lag-time values. However, in lag-time TTO, more people were willing to trade off time for mild states, though less time on average (i.e., higher mean values) than in lead-time TTO. Thus, the findings were mixed regarding the effect of the specification of TTO on health-state values.

A 1995 study into time preferences and the duration of health states by Dolan and Gudex [11] compared lead-time TTO with lag-time TTO, but without using those exact terms for the TTO specifications. That study had a lead-time TTO and a lag-time TTO with 9 years in full health and 1 year in an impaired health state. For three out of five health states, lead-time median values were lower than lag-time values. Thus, for three out of five health states, respondents considered having the health impairment earlier preferable to having it later (i.e., negative time preferences). Although this finding seemingly contradicts the results presented here, it may well be that individuals obtain more utility from having the health impairment earlier when the duration of the health state is relatively short; that is, they might prefer to get the poor health state ‘over with.’ This reasoning would be in line with our finding that for the shorter disease duration the difference between lead-time TTO and lag-time TTO is smaller. These results highlight the influence of time preference in TTO tasks, especially when the addition of lead or lag-time increases the considered time horizon. A detailed study into correcting the TTO values from this study for time preferences is currently underway.

The relative importance of different domains was affected by the duration of the health state in the experiment. Although all of the variants tested indicated that the domains ‘pain/discomfort’ and ‘anxiety/depression’ caused the largest decrement in health state utilities, the ‘anxiety/depression’ domain was given more weight for longer durations in all three TTO tasks. If the relative importance of an attribute of a health state depends on its duration, it is unlikely that the specific health-state value decrement can be extrapolated to durations other than the one applied in the TTO task.

Although the instructions for the online TTO were very carefully designed by a team of researchers with experience in TTO, and even though the respondents were given both textual and graphical explanations, the level of task engagement was low in the online setting. Roughly two-thirds of the observations used a maximum of four iterations to achieve the point of indifference. With the iteration procedure applied in this study, this means that two-thirds of the health states were valued at either 1 (one iteration), 0 (two iterations), 0.5/−0.5 (three iterations), or 0.6/−0.6/0.4/−0.4 (four iterations). It is possible that the respondents did not know their preference more precisely than that represented by one of these health-state values. Yet perhaps the level of task engagement could be improved by a different mode of administration. For example, the median number of iterations for classic TTO in a face-to-face interview setting, as reported elsewhere [16], was seven, compared to three in this study. Indeed, TTO data collection via the Internet may produce lower data quality for classic TTO [17], although it has also been argued that it facilitates a good geographical coverage of respondents at a low cost [18]. Nonetheless, a comparison of our online study with results from face-to-face interviews does highlight some differences. In a previous Dutch valuation study of EQ-5D-3L, using classic TTO, the value of the worst health state (33333) was −0.39 and that of the second best health state (11211) was 0.897 [19]. That range was not reflected in any of the TTO specifications tested here. Excluding participants who claimed not to understand the task, those respondents who did not differentiate between health states or used less than three iterations did not alter this finding. Similarly, the health-state values of the classic TTO, with a transformation for negative values to be bound at −1 as applied in the previous TTO valuation studies of EQ-5D-3L, did not produce negative mean values for any of the health states. Thus, classic TTO also had a rather limited range of values compared to previous EQ-5D valuation studies [19, 20]. Unlike Devlin et al., we did not find notably less non-trading for mild states in lag-time TTO compared to lead-time TTO [8].

Heterogeneity was greatest for lag-time TTO variants, suggesting that respondents’ answers differ more in this task than in classic TTO or lead-time TTO, which could be due to several unknown variables. These results seem to indicate that respondents were better able to grasp the lead-time TTO task, leading to less difference in answers. Yet such a conclusion would not fully align with the self-reported feasibility of the task. The latter indicates that lead-time TTO was, on average, considered slightly more difficult than lag-time TTO. The increased variance in the lag-time TTO tasks is thus not solely attributable to understanding of the task.

## Conclusion

Lead-time TTO and lag-time TTO yield different health-state values. Differences between lead-time TTO and lag-time TTO seem to be systematic, an observation that requires further study.

## Appendix

See Table 3.

**Table 3:** Checklist to compare TTO specifications

*Methodological*	
Type of TTO method	Lead-time TTO and lag-time TTO
Incorporated discounting?	No
Total time frame in years	Lead-time TTO: 15 and 20, Lag-time TTO: 15 and 20
Health-state duration	5 and 10 years
Lead-time length	10 years
Lag-time length	10 years
Ratio of lead/lag-time to health state duration	2:1 and 1:1
Lowest possible value	−1 and −2
Who valued the health state?	General population
Description of health state at which value = 1	Full health
Worst health state	55555
What was valued?	EQ5D-5L
Health state selection	D-optimal design
Blocked design?	Yes
Number of health states	100
Health states per respondent	10
Sample size	5,208
Valuations per health state	About 100
*Procedural*	
TTO procedure	Structured iteration
Iteration first question	Value = 1
Iteration second question	Value = 0
Number of attributes	5
Levels per attribute	5
Highest attainable value	1
Warm-up tasks	Discrete choice experiment with similar health states
Visual representation	Side-by-side presentation of alternatives
Interviewer interaction with protocol standardized?	Yes
Mode of administration	Online interviews
Smallest tradable unit	3 months
WTD procedure	Not applicable
*Analytical*	
Transformation of WTD	Not applicable
Exclusion criteria	Respondent who did not complete the task

## Footnotes

1 Negative values transformed with −*x/*(*t−x*).
